# The Systemic Inflammation Response Index as an Independent Predictor of Acute Kidney Injury in Critically Ill Patients With Acute Myocardial Infarction: Insights From a Large-Scale Cohort Study

**DOI:** 10.1155/mi/1417075

**Published:** 2025-11-21

**Authors:** Xudong Li, Liang Ruan, Shuyuan Zhang, Yuhan Qin, Yang Pu, Jianing Yang, Shuailei Xu, Huihong Tang, Chengchun Tang, Yong Qiao

**Affiliations:** ^1^Department of Cardiology, Zhongda Hospital, Southeast University, Nanjing, Jiangsu, China; ^2^School of Medicine, Southeast University, Nanjing, Jiangsu, China; ^3^School of Clinical Medicine, Xinjiang Second Medical College, Karamay, Xinjiang, China

**Keywords:** acute kidney injury, acute myocardial infarction, critical care, inflammation, Systemic inflammation response index

## Abstract

**Background:**

Acute kidney injury (AKI) is a critical risk factor for adverse outcomes in acute myocardial infarction (AMI) patients admitted to the intensive care unit (ICU). Early identification of high-risk patients is essential for personalized treatment. The systemic inflammation response index (SIRI), a marker of systemic inflammation, has not been fully explored for its predictive role in AKI.

**Methods:**

This study included 6936 critically ill AMI patients from the MIMIC-III and MIMIC-IV databases Lasso regression, multivariate logistic regression, restricted cubic spline (RCS) models, and subgroup analyses were employed to explore the association between SIRI and AKI risk. Then, we constructed a predictive model based on these findings internally validated using bootstrapping (1000 repetitions). Discrimination was assessed by the optimism-corrected area under the receiver operating characteristic (ROC) curve (areas under the curve [AUC]), and calibration was evaluated by the calibration curve and the Hosmer–Lemeshow test. The optimal cutoff value for SIRI was determined using the Youden index and propensity score matching (PSM; 1:1) was performed. Conditional logistic regression was used to validate the robustness of this association. Additionally, Cox regression and Kaplan–Meier survival analyses were conducted to assess the relationship between SIRI and in-hospital mortality in the overall cohort.

**Results:**

Elevated SIRI levels independently predicted AKI, showing a nonlinear relationship. Subgroup and propensity-matched analyses confirmed this association. Furthermore, the predictive performance of the model was robust upon internal validation. The optimism-corrected AUC was 0.767 (95% CI: 0.755–0.781) and the calibration curve showed excellent agreement, the Hosmer–Lemeshow test indicated good fit (*p*=0.539). Kaplan–Meier curves revealed higher in-hospital mortality in higher SIRI quartiles (log-rank *p*  < 0.001). Multivariate Cox regression further supported SIRI as a significant predictor of in-hospital mortality.

**Conclusion:**

SIRI is an independent risk factor for AKI and in-hospital mortality in critically ill AMI patients, offering valuable clinical utility for early AKI prediction and risk stratification.

## 1. Introduction

Acute kidney injury (AKI) is a common and critical condition in clinical practice, studies have reported that 10%–15% of patients in general wards have AKI, while more than half of the patients in intensive care units (ICUs) suffer from it, significantly affecting their quality of life [1, 2]. After acute myocardial infarction (AMI), due to factors such as hemodynamic changes and activation of the sympathetic nervous system, the kidneys are susceptible to acute injury. This often leads to increased healthcare costs and poor prognosis. Although significant progress has been made in the treatment of AMI, the incidence of AKI remains high, particularly among critically ill patients admitted to ICUs [3]. In this population, identifying modifiable risk factors for AKI is crucial for early intervention and improving prognosis.

The inflammatory response following AMI is characterized by the activation of immune cells and the release of pro-inflammatory cytokines such as tumor necrosis factor-*α* (TNF), interleukin-1*β* (IL-1*β*), and IL-6 [4]. Myocardial ischemia can trigger an inflammatory cascade, recruiting circulating neutrophils and pro-inflammatory monocytes mobilized from the spleen and bone marrow, systemic inflammation can lead to renal vascular dysfunction and damage to renal parenchymal cells, making it prone to rapid decline in kidney function, resulting in the development of AKI [5, 6]. Previous studies [7] have shown that dendritic cells, monocytes/macrophages, neutrophils, T lymphocytes, and B lymphocytes play crucial roles in the development of AKI.

In recent years, composite indices derived from complete blood count have gradually gained attention as potential indicators of systemic inflammation and immune dysregulation. The systemic inflammation response index (SIRI) is an emerging and reliable indicator that reflects systemic inflammation. It is defined as the product of neutrophil count and monocyte count divided by the lymphocyte count [8]. SIRI integrates information from three key components of the immune system and has been shown to be associated with poor prognosis in various cardiovascular and renal diseases [9–13]. However, its role as a predictor of AKI in critically ill patients with AMI has not been fully investigated. This study aims to investigate the association between the SIRI and the incidence of AKI in the patients by analyzing retrospective cohort data, providing an easily accessible biomarker for risk stratification in this patient population. The findings may be of significant importance for early identification of high-risk patients and for developing targeted therapeutic strategies to reduce the risk of AKI.

## 2. Methods

### 2.1. Study Design and Population

**Figure 1:**
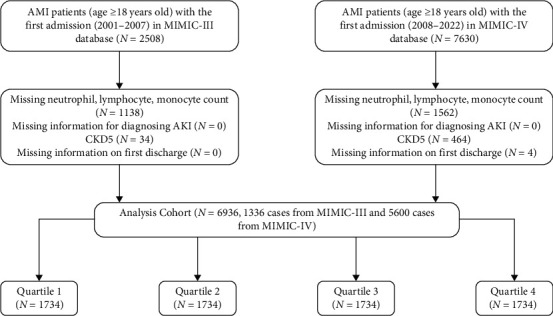
Study flowchart of patient selection. Quartile 1: SIRI ≤ 1.68; Quartile 2: 1.68 < SIRI ≤ 3.51; Quartile 3: 3.51 < SIRI ≤ 7.62; Quartile 4: SIRI > 7.62.

This retrospective study included patients aged ≥18 years who were admitted to the ICU due to AMI between 2001 and 2022, using data from the MIMIC-III (version 1.4) and MIMIC-IV (version 3.0) datasets. In cases of repeated hospitalizations, only the data from the first hospitalization were considered. The study excluded participants based on the following criteria: (1) missing essential data (neutrophil, lymphocyte, and monocyte counts); (2) absence of a definitive AKI diagnosis; (3) admission to the ICU with Stage 5 chronic kidney disease (CKD); (4) missing discharge information (Figure 1). We divided the study population into four groups based on quartiles of SIRI (cut points set at 25%, 50%, and 75%).

The MIMIC dataset is a collaboration between Beth Israel Deaconess Medical Center (BIDMC) and the Massachusetts Institute of Technology (MIT). One of the authors of this study (Liang Ruan) completed the necessary training and obtained permission to use the dataset (certification number: 60092717). Since the two datasets were approved for exemption from informed consent and sharing, no informed consent was required from the patients for this study.

In this study, the diagnoses of AMI, acute heart failure (AHF), hypertension, atrial fibrillation (AF), type 2 diabetes mellitus (T2DM), CKD, and cerebrovascular disease (CD) were established using the International Classification of Diseases (ICD-9 and ICD-10) coding system.

### 2.2. Data Collection

In this study, data were extracted using Structured Query Language (SQL). The collected data encompassed demographic characteristics, comorbidities, history of coronary artery surgery, sequential organ failure assessment (SOFA) scores, laboratory test results, prescribed medications, and discharge-related information. All laboratory test results were derived from the initial measurements obtained at the time of patient admission and urine output (UO) was extracted as the total urine volume within the first 24 h after admission.

SIRI is defined as the product of the neutrophil count and monocyte count divided by the lymphocyte count. It is calculated using the following formula: SIRI = (neutrophil count × monocyte count)/lymphocyte count.

### 2.3. Clinical Outcomes

The occurrence of AKI was considered the primary endpoint. (1) Serum creatinine (Scr) increased ≥1.5 times from baseline within the past 7 days; (2) Scr increased ≥0.3 mg/dL (26.5 *μ* mol/L) within 48 h; (3) UO was less than 0.5 mL/kg/h for a continuous period of 6 h. According to the Kidney Disease: Improving Global Outcomes (KDIGO) guidelines [14], AKI is diagnosed if any of the above criteria are met. The secondary endpoint was defined as in-hospital all-cause mortality in critically ill AMI patients and those with concomitant AKI.

### 2.4. Statistical Analysis

**Figure 2:**
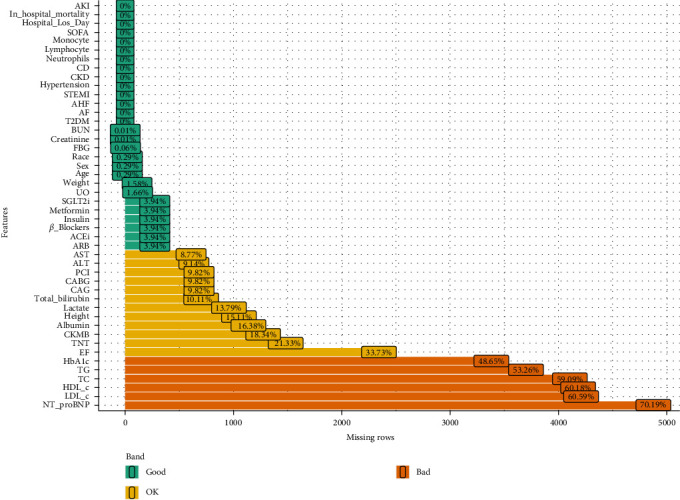
Proportion of missing data for variables. Good (green): 0% missing values, OK (yellow): missing values not exceeding 40%; bad (orange): missing values exceeding 40%.

Data with missing values exceeding 30% were excluded, and variables with missing data less than 30% were imputed using the “missForest” package in R (Figure 2). The imputation process showed good convergence, with the normalized root mean square error (NRMSE) decreasing from 0.158 to 0.150 and the proportion of falsely classified (PFC) values stabilizing at 0.0875, indicating robust performance (Figure S1). The cohort was divided into two groups based on the occurrence of AKI. Normality tests were conducted, with continuous variables that followed a normal distribution presented as mean (standard deviation, SD) and those with skewed distribution presented as median (interquartile range [IQR]). Intergroup comparisons were performed using the Kruskal–Wallis *H* test, Student *t*-test, or one-way analysis of variance (ANOVA). Categorical variables were expressed as frequencies and percentages (%) and compared using the Chi-square test or Fisher's exact test.

Receiver operating characteristic (ROC) curves were employed to evaluate the predictive value of the SIRI and established white blood cell-derived inflammatory indices for AKI, including the monocyte to lymphocyte ratio (MLR), neutrophil to lymphocyte ratio (NLR), and neutrophil to monocyte plus lymphocyte ratio (NMLR). The areas under the curves (AUCs) were compared using DeLong's test.

**Figure 3:**
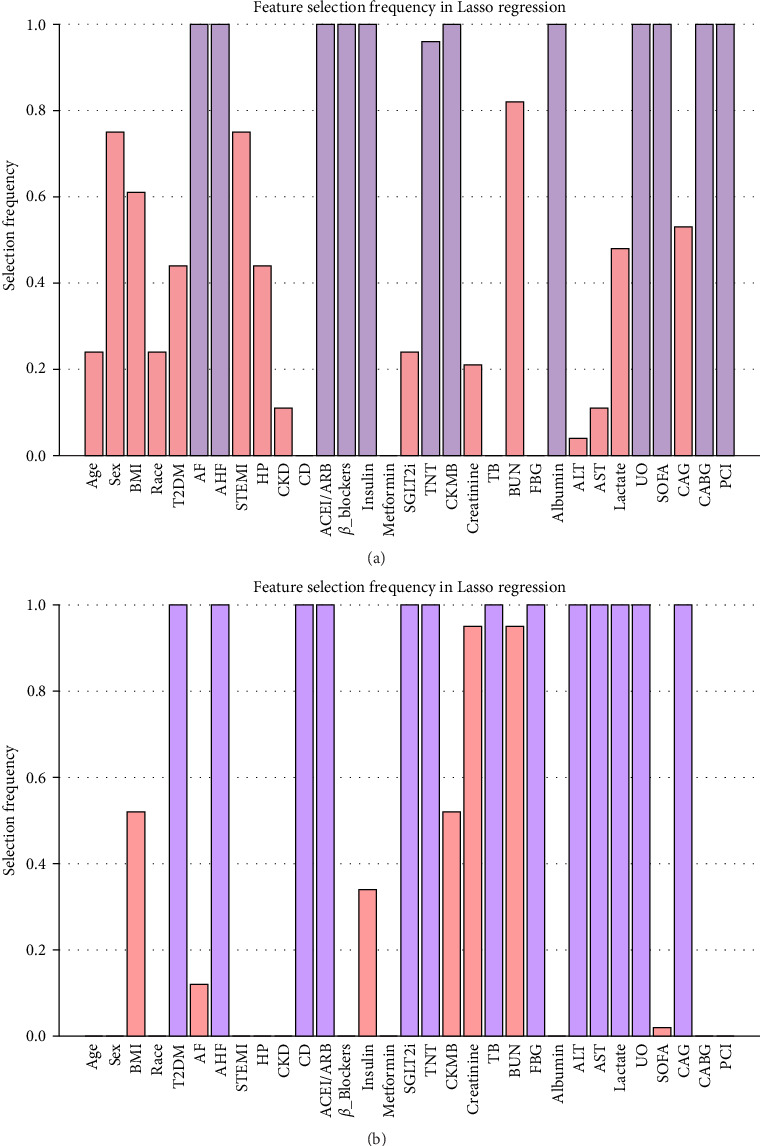
Feature selection frequency in Lasso regression (A) for occurrence of AKI and (B) in-hospital mortality. Purple denotes regions with selection frequency ≥0.95, and pink denotes those <0.95.

**Figure 4:**
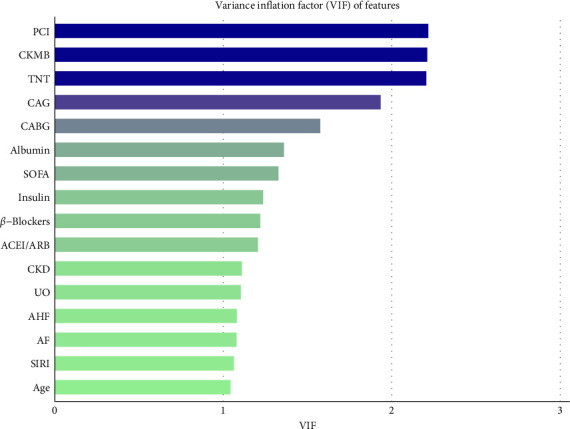
Variance inflation factor (VIF) of features.

To assess the correlation between SIRI and the risk of AKI in critically ill AMI patients, variables including age, sex, body mass index (BMI), T2DM, AF, AHF, ST-segment elevation myocardial infarction (STEMI), hypertension, CKD, CD, angiotensin-converting enzyme inhibitors/angiotensin receptor blockers (ACEIs/ARBs), *β*-blockers, metformin, insulin, sodium-dependent glucose transporter 2 inhibitor (SGLT2i), troponin T (TNT), creatine kinase-myocardial band (CKMB), creatinine, total bilirubin (TB), blood urea nitrogen (BUN), fasting blood glucose (FBG), albumin, alanine aminotransferase (ALT), aspartate aminotransferase (AST), lactate, UO, SOFA scores, coronary angiography (CAG), coronary artery bypass grafting (CABG), and percutaneous coronary intervention (PCI) were selected using Lasso regression. During the Lasso regression cross-validation, “lambda.1se” was selected as the *λ* value (the maximum *λ* value within one standard error of the minimum error), and to enhance Lasso regression stability, 100 iterations of Lasso regression were run, with the frequency of selection of each feature being recorded. Features with a selection frequency ≥0.95 were deemed stable and important variables for further analysis (Figure 3) [15, 16]. We then assessed multicollinearity among the variables selected through the above process, as well as clinically relevant variables including age, CKD, and CAG, using the variance inflation factor (VIF). A VIF ≥ 5 was considered indicative of multicollinearity. None of these variables exhibited multicollinearity (Figure 4). Model 1 was unadjusted, Model 2 adjusted for age, sex, race and BMI, and Model 3 adjusted for age, AF, AHF, CKD, ACEI/ARB, *β*-blockers, insulin, TNT, CKMB, albumin, UO, SOFA, CAG, CABG, and PCI. Odds ratios (ORs) and their corresponding 95% confidence intervals (CIs) were calculated to quantify the effect of the SIRI index on these outcomes.

To explore the dose–response relationship between SIRI and AKI, restricted cubic spline (RCS) plots were drawn in Model 1, Model 2, and Model 3, with knots at the 5th, 35th, 65th, and 95th percentiles. Additionally, age, sex, T2DM, STEMI, and CKD were used as subgroups for subgroup analysis.

Given the lack of an external validation cohort for SIRI plus Model 3, we employed the bootstrap resampling method for internal validation of the model. This involved performing repeated random sampling with replacement (*B* = 1000 times) from the original dataset. Model performance was primarily evaluated in terms of discrimination and calibration. Discrimination was quantified using the AUC, and the optimism-corrected AUC along with its 95% CI are reported. Calibration was comprehensively assessed using the calibration curve and the Hosmer–Lemeshow test. The calibration curve visually illustrates the agreement between predicted risk and observed risk. The null hypothesis of the Hosmer–Lemeshow test is that the model predictions perfectly fit the observed values. Furthermore, to evaluate the clinical net benefit of the model across different decision thresholds, we conducted a decision curve analysis (DCA). Subsequently, the net reclassification improvement (NRI) and integrated discrimination improvement (IDI) were calculated using the “PredictABEL” package in R to quantify the incremental predictive value of SIRI for AKI.

Sensitivity analysis was conducted to verify the robustness of the results. First, to ensure a complete 48-h observation window for diagnosing AKI and to reduce confounding effects due to early death or transfer, logistic regression analysis was performed after excluding patients admitted to the ICU for less than 48 h (285 patients excluded) from the entire cohort. Second, the “pROC” package in R was used to determine the cutoff value of SIRI based on the optimal Youden index. The entire cohort was then divided into two groups for 1:1 propensity score matching (PSM) analysis. Variables with *p*  < 0.05 between the two groups were matched using the nearest neighbor method, with a caliper value of 0.1, and a standardized mean difference (SMD) <0.1 was considered indicative of good balance between the groups [17]. Conditional logistic regression was used to analyze the potential relationship between SIRI and AKI.

The Cox proportional hazards model was used to explore the relationship between SIRI and in-hospital mortality. All variables were entered into Lasso regression (method as described above). Ultimately, T2DM, AHF, CD, ACEI/ARB, SGLT2i, TNT, TB, FPG, ALT, AST, lactate, UO, and CAG were selected (Figure 3B). Clinically relevant variables—age, BMI, and albumin—along with the statistically significant variables identified above, were included in Model C after confirming the absence of multicollinearity (all VIFs < 5). Model A was unadjusted for confounding factors, while Model B was adjusted for age, sex, race, and BMI. Additionally, to account for the potential influence of AKI on in-hospital mortality, we performed a sensitivity analysis. Patients who died within 48 h were excluded, and 48 h AKI status was incorporated as a covariate in Model C for Cox regression analysis to examine the robustness of the association between SIRI and in-hospital mortality. To investigate the heterogeneous effect of SIRI on mortality between AKI and non-AKI populations, we performed a stratified analysis based on AKI occurrence within Model C and assessed whether an interaction exists between SIRI and AKI. Hazard ratios (HRs) and their corresponding 95% CIs were calculated. Additionally, Kaplan–Meier survival curves were plotted to examine differences in in-hospital mortality between different quartiles of SIRI, and the Log-rank test was used to assess inter-group differences.

All data analyses were performed using R version 4.4.2 (R Foundation for Statistical Computing, Vienna, Austria). A two-sided *p*-value <0.05 was considered statistically significant.

## 3. Results

### 3.1. Baseline Characteristics

As shown in Table 1, a total of 5430 patients (78.3%) in the cohort developed AKI, while 1506 patients (21.7%) did not. Among the 6936 patients, 1060 individuals (15.3%) experienced in-hospital mortality. The study found that patients who developed AKI were older, more likely to have AF, AHF, and CKD, and had a higher proportion of receiving CABG treatment. Compared to patients who did not develop AKI, those with AKI had higher neutrophil counts, creatinine, BNU, ALT, AST levels, and SOFA scores upon admission. Conversely, they had lower albumin levels, 24 h UO. Additionally, AKI patients had longer hospital stays and a higher in-hospital mortality rate.

Study participants were categorized into four groups based on SIRI quartiles: Quartile 1 (SIRI ≤ 1.68), Quartile 2 (1.68 < SIRI ≤ 3.51), Quartile 3 (3.51 < SIRI ≤ 7.62), and Quartile 4 (SIRI > 7.62). We compared the incidence of AKI, in-hospital mortality and length of hospital stay among different SIRI quartiles (Table 2). The results showed statistically significant differences across the four groups in terms of AKI incidence, in-hospital mortality, and length of hospital stay (all *p*  < 0.05). The highest SIRI quartile had the highest rates of AKI, in-hospital mortality, and longer hospital stays.

### 3.2. Correlation Between SIRI and the Occurrence of AKI

ROC analysis demonstrated comparable predictive performance of SIRI, MLR, NLR, and NMLR for AKI, with AUC of 0.584, 0.578, 0.583, and 0.583, respectively (Table S1). DeLong's test indicated no statistically significant differences in AUC between SIRI and each of the other inflammatory indices (all *p* > 0.05).

**Figure 5:**
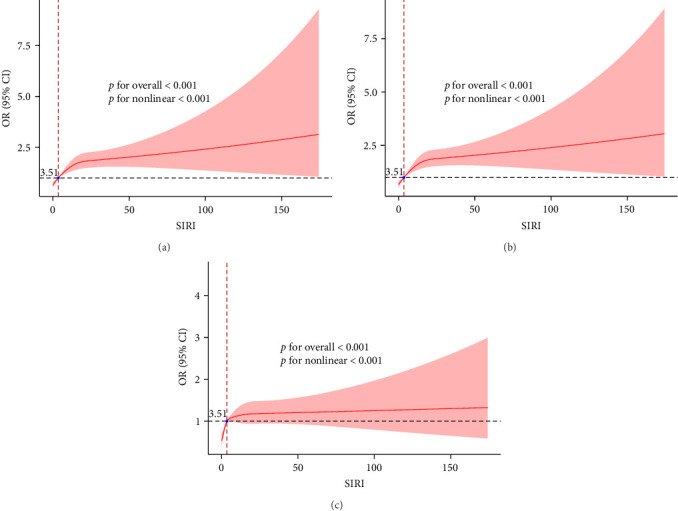
Restricted cubic spline for correlation between SIRI and the occurrence of AKI. (A) Model 1 was unadjusted. (B) Model 2 was adjusted for sex, race, age, and BMI. (C) Model 3 was adjusted for age, AF, AHF, CKD, ACEI/ARB, *β*-blockers, insulin, TNT, CKMB, albumin, UO, SOFA, CAG, CABG, and PCI.

As shown in Table 3, logistic regression analyses revealed that, using the lowest SIRI quartile as a reference, higher levels of SIRI were consistently associated with the occurrence of AKI across all three models. In the fully adjusted model (Model 3), the risk of AKI in the highest SIRI quartile was 1.76 times that of the lowest quartile (Q4 vs. Q1: OR = 1.76 [95% CI: 1.44, 2.15], *p*  < 0.001; Table S2). RCS were used to explore the nonlinear relationship between SIRI and AKI occurrence in critically ill AMI patients. As shown in Figure 5, a nonlinear relationship was found between SIRI and the occurrence of AKI (all *p* for nonlinear <0.05). When SIRI > 3.51 (approximate), excessively high SIRI became a risk factor for AKI, with the risk of AKI continuing to increase.

**Figure 6:**
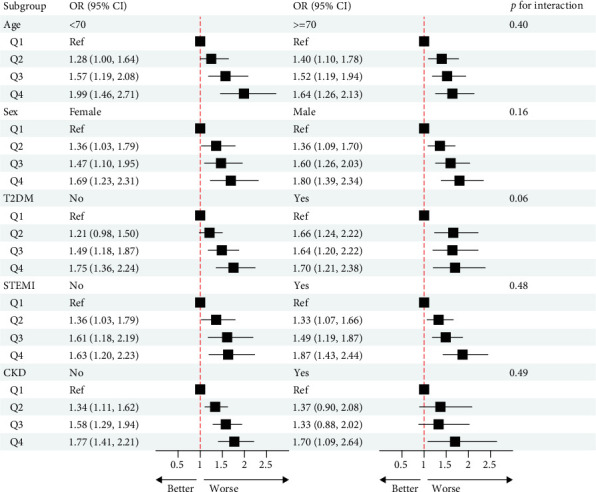
Forest plot for the associations of SIRI and AKI occurrence in different subgroups. CKD, chronic kidney disease; STEMI, ST-segment elevation myocardial infarction; T2DM, type 2 diabetes mellitus.

Subgroup analysis (Figure 6) demonstrated that in the subgroups of age <70, age ≥70, female, male, with T2DM, without T2DM, STEMI, NSTEMI, with CKD, and without CKD, SIRI was an independent risk factor for AKI in critically ill AMI patients after full adjustment for confounding factors, and no interaction between SIRI and subgroup variables was found. Notably, in without CKD subgroup, compared to Q1, the second, third, and fourth quartiles of SIRI were associated with the occurrence of AKI, whereas in the with-CKD subgroup, only the highest SIRI quartile showed a statistically significant correlation with AKI occurrence.

The internal bootstrap validation results (with 1000 repetitions) indicated that the aforementioned Model 3 exhibited favorable performance (Figure S2). In terms of discrimination, the optimism-corrected AUC for predicting AKI was 0.767 (95% CI: 0.755–0.781). Regarding calibration, the calibration curve demonstrated that the corrected predicted risks were highly consistent with the actual observed risks, with the curve closely aligning with the ideal diagonal line. Calibration analysis revealed a calibration slope of 1.0009 and an optimism-corrected intercept of −0.0007, with a corresponding optimism bias of −0.0006. The Hosmer–Lemeshow test yielded a *p*-value of 0.539, indicating no statistical evidence to reject the null hypothesis of good model calibration and supporting a strong agreement between predicted and observed risks. DCA suggested potential clinical utility of the model. The incorporation of SIRI into Model 3 resulted in statistically significant improvements in both reclassification and discrimination (Table S3). The continuous NRI was 19.84% (95% CI: 14.16–25.53%; *p*  < 0.001). The IDI reached 0.54% (95% CI: 0.34–0.73%; *p*  < 0.001), confirming enhanced overall model performance.

**Figure 7:**
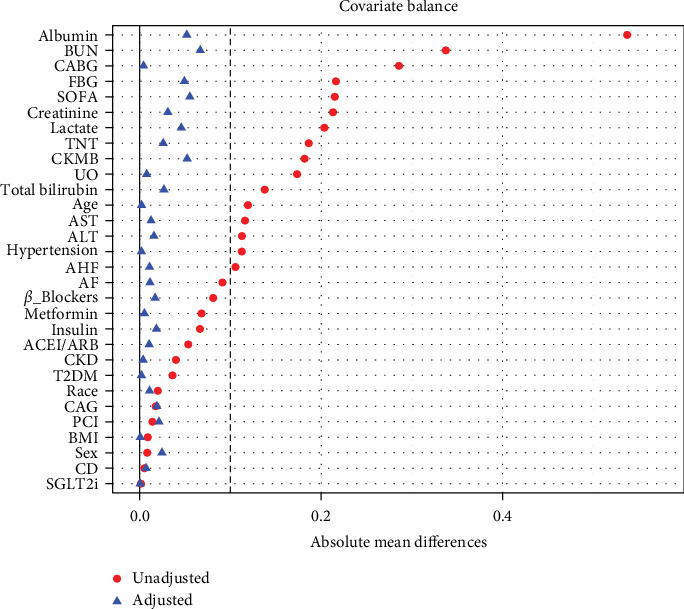
Covariate balance assessment before and after PSM.

Furthermore, logistic regression analysis excluding patients who were admitted to the ICU for less than 48 h produced results consistent with the previous logistic analysis (Table 4). As the SIRI quartiles increased, the risk of AKI also gradually increased. We further determined the cutoff value of SIRI = 2.707 based on the optimal Youden index and divided the cohort into two groups for PSM based on this cutoff (Table S4 and S5). The baseline characteristics of the matched cohort were well balanced, with all SMDs <0.1 (Figure 7 and Table S6). Conditional logistic regression was then performed on the matched cohort (Table 5). After fully adjusting for confounding factors, the risk of AKI in patients with SIRI ≥2.707 was 47% higher than that in patients with SIRI <2.707. Additionally, we also performed conditional logistic regression using the SIRI quartiles from the original cohort (Table S7) and found that the risk of AKI in the highest SIRI quartile was 78% higher than in the lowest quartile (OR = 1.78 [95% CI: 1.38–2.29]). In the matched cohort, the risk of AKI in the highest SIRI quartile was higher compared to the original cohort (OR = 1.78 [95% CI: 1.38–2.29] vs. 1.76 [95% CI: 1.44–2.15]).

### 3.3. Correlation Between SIRI and In-Hospital Mortality

**Figure 8:**
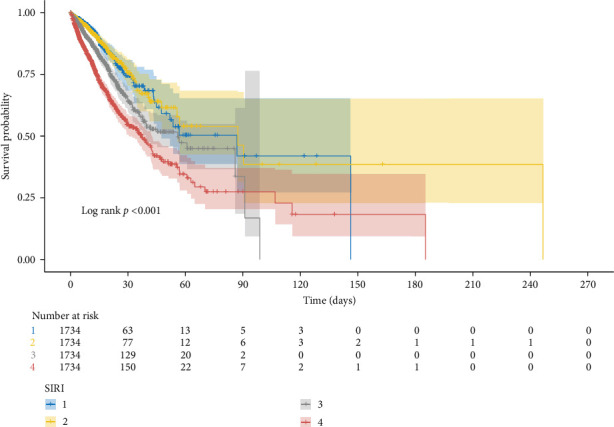
Kaplan–Meier survival curves stratified by SIRI quartiles in whole population.

The Kaplan–Meier survival curve (Figure 8) demonstrated statistically significant differences in in-hospital mortality across different SIRI quartiles in the entire cohort (log-rank *p*  < 0.001). Multivariate Cox regression analysis (Table 6) showed that SIRI was significantly associated with in-hospital mortality. Compared to Q1, the risk of in-hospital mortality in patients in the Q4 group increased by 74%(*p*  < 0.05; Table S8). Sensitivity analysis (Table S9) indicated that SIRI remained statistically significantly associated with in-hospital mortality even after adjusting for AKI as a covariate. After stratification by AKI status (Table 7), we found that this association was only observed in the population with AKI, while no statistically significant correlation was found in those without AKI (all *p* > 0.05). However, the test for interaction did not show statistical significance (*p* for interaction = 0.214). This may indicate that the modifying effect of AKI status on the association between SIRI and in-hospital mortality did not reach statistical significance.

## 4. Discussion

In a retrospective cohort study conducted on a U.S. population, we identified that: (1) SIRI is an independent risk factor for the increased occurrence of AKI in critically ill AMI patients, showing a nonlinear relationship and (2) SIRI serves as an independent predictor of in-hospital mortality in critically ill AMI patients. Notably, this study highlights that SIRI can efficiently and easily identify individuals at high risk for AKI early in ICU patients with AMI.

SIRI is a reliable indicator of systemic inflammation, integrating the three major components of peripheral blood leukocytes: neutrophils, lymphocytes, and monocytes. Previous studies have confirmed that SIRI is associated with the occurrence, development, and prognosis of various diseases. Two large prospective cohort studies, one [18] involving 42,875 U.S. adults followed for 20 years and the other [19] with 85,154 Chinese adults followed for 10 years, demonstrated that higher baseline SIRI levels increased the risk of long-term cardiovascular and all-cause mortality. Additionally, SIRI has been shown to correlate with the occurrence of acute coronary syndrome (ACS), the severity of coronary artery disease (CAD), and poor long-term prognosis [8, 20]. Furthermore, several studies [13, 21, 22] have confirmed that SIRI is an independent predictor of AKI in various patient populations, such as those undergoing elective PCI, abdominal trauma patients, and critically ill pediatric ICU patients. However, there has been no study exploring the potential relationship between SIRI and the risk of AKI in ICU patients with AMI. This study, through LASSO regression and multivariate logistic regression, demonstrated that SIRI is a robust and strong predictor of AKI occurrence risk after adjusting for potential confounding factors. After excluding patients with an ICU stay of less than 48 h, the association between SIRI and AKI remained statistically significant with a comparable effect size, indicating that the study conclusions were not influenced by short-term hospitalizations. This result reinforces the generalizability of SIRI as an early predictive biomarker, demonstrating that its predictive value remains reliable even when a sufficient observation window is ensured.

The pathogenesis of AKI following AMI is complex and not fully understood; however, inflammation plays a critical role. AMI can lead to AHF and decreased cardiac output, and the kidneys, which receive 25% of cardiac output, are particularly vulnerable to these hemodynamic changes, leading to ischemia-reperfusion injury. Damaged endothelial cells release cytokines and chemokines, leading to the infiltration of various inflammatory cells into the renal interstitium [23, 24]. Moreover, AMI induces a stress response that triggers an inflammatory cascade, recruiting circulating neutrophils and pro-inflammatory monocytes mobilized from the spleen and bone marrow. These immune cells interact with renal parenchymal cells, causing renal damage [5, 7]. Specifically, neutrophils can infiltrate the kidneys early in injury, promoting the secretion of proteases and reactive oxygen species (ROS), which in turn damage the endothelial cells [25]. Furthermore, animal studies [26] have shown that the decreased infiltration of neutrophils was linked to maintained renal function and structural integrity. Several subtypes of lymphocytes, such as B lymphocytes, CD8 T lymphocytes and T helper 1 (Th1), are involved in AKI development, while T helper 2 (Th2) and regulatory T cells (Tregs) play protective roles due to their anti-inflammatory properties [24]. Previous research has shown that early neutrophil recruitment and the secretion of ROS and other inflammatory factors promote the transformation of monocytes into pro-inflammatory macrophages. These macrophages release pro-inflammatory cytokines, such as IL-6, which contribute to tubular damage [27]. Notably, the release of pro-inflammatory cytokines into the bloodstream can suppress cardiac function, manifesting as a decrease in ejection fraction. The complex bidirectional relationship between the heart and kidneys is commonly referred to as cardiorenal syndrome (CRS) [23]. The potential mechanisms described above are supported by the findings of our study. We performed univariate logistic regression analyses to examine the association between each component of SIRI and AKI. As shown in Table S10, both neutrophil count and monocyte count were identified as risk factors for AKI (OR = 1.04 [95% CI: 1.02–1.05] and OR = 1.30 [95% CI: 1.15–1.46], respectively). In contrast, no statistically significant harmful effect was observed for peripheral lymphocyte count (OR = 0.96 [95% CI: 0.94–0.99]). These findings may provide empirical support for the proposed mechanisms.

We also observed that 24.00% of AKI patients had CKD at ICU admission, while only 17.20% of non-AKI patients had CKD (*p*  < 0.05). CKD is considered a chronic inflammatory condition, characterized by elevated pro-inflammatory cytokines (such as IL-1, IL-6, and TNF-*α*), increased oxidative stress, and decreased antioxidant capacity, which makes these patients more susceptible to acute kidney dysfunction [28, 29]. It is worth noting that subgroup analysis showed that in CKD patients, the correlation between SIRI and AKI was only significant in the highest SIRI quartile (*p*=0.019), whereas in non-CKD patients, this correlation existed in all other SIRI quartiles (all *p*  < 0.05). This finding is somewhat consistent with a clinical study [21] that included 5685 patients undergoing elective PCI and examined the correlation between SIRI and contrast-induced nephropathy. The study showed that the correlation only existed in patients with an eGFR <60 mL/min/1.73 m^2^ upon admission. This may be because the baseline inflammatory state in CKD patients results in a higher threshold for inflammatory responses. They may be more tolerant to mild or moderate levels of inflammation, and only when SIRI reaches higher levels does the inflammation burden increase, leading to AKI. On the other hand, kidneys in a physiological state are more sensitive to inflammation, and even mild or moderate levels of inflammation can lead to AKI.

Increasing evidence shows that elevated markers of systemic inflammation are predictive of poor clinical outcomes, including death and recurrent myocardial infarction in AMI patients. A study [30] involving 4291 AMI patients admitted to the ICU demonstrated that SIRI can predict 30- and 90-day all-cause mortality. Other studies [31] have also shown that combining SIRI with traditional cardiovascular risk factors improves the prediction of in-hospital mortality risk in AMI patients. Our study suggests that higher levels of SIRI are an independent predictor of in-hospital mortality in critically ill AMI patients. After AMI, neutrophil infiltration into coronary plaques and infarcted myocardium leads to tissue damage mediated by the release of matrix-degrading enzymes and ROS. Monocytes and macrophages are important contributors to atherosclerotic plaque formation and can promote inflammation [32]. Research [33] has shown that elevated peripheral blood monocytes after AMI are associated with adverse left ventricular remodeling and poor cardiovascular events. Cox regression analysis also indicated that a lower lymphocyte count is associated with adverse cardiovascular events in STEMI patients [34]. After AMI, Tregs infiltrating myocardial tissue can repair the heart by inhibiting inflammatory cell recruitment, improving left ventricular remodeling, and reducing myocardial cell apoptosis [35–37].

Currently, patients with the dual burden of heart and kidney diseases still exhibit high rates of hospitalization and mortality, along with a lower quality of life. Therefore, early identification of high-risk AMI populations prone to developing AKI holds significant clinical importance. SIRI, which can be calculated from routine blood tests, is characterized by its low cost and ease of acquisition, providing additional value for clinical diagnosis and treatment. For AMI patients with elevated SIRI levels, vigilance is warranted regarding the development or progression of AKI. Nephrotoxic medications should be avoided, and the use of contrast agents minimized. Fluid intake and output should be carefully managed to alleviate renal congestion or prevent hypoperfusion. Regular monitoring of indicators such as creatinine, urea, and UO is essential to avoid missed or delayed diagnosis, which could postpone AKI treatment. Additionally, underlying conditions should be actively managed, including correcting anemia and controlling blood glucose and blood pressure [38, 39]. Our study is the first clinical research to explore the relationship between SIRI and the occurrence and prognosis of AKI in AMI patients admitted to the ICU, filling this gap. SIRI, which can be calculated from routine blood tests, is characterized by its low cost and ease of acquisition, providing additional value for clinical diagnosis and treatment.

## 5. Strengths and Limitations

First, as a retrospective cohort study, this research cannot establish a causal relationship between SIRI and AKI. To enhance the reliability of the results, we included as many samples as possible (6936 cases). Additionally, due to the inherent limitations of the database, some potential confounding factors, such as the duration of CKD and prior medication use, were not considered in this study, introducing some uncertainty into the findings. However, for the available and known confounders, we employed univariate and multivariate logistic regression, Lasso regression, and PSM to minimize the impact of confounding factors on the results. Last, while our findings are robust within the MIMIC-III/IV cohorts, the generalizability to other populations (e.g., non-U.S. or non-ICU settings) requires further validation. Future studies should include external cohorts from diverse geographic and clinical backgrounds to confirm the universal applicability of SIRI. Our study is the first to investigate the relationship between SIRI and the occurrence of AKI in critically ill AMI patients, and more prospective studies are needed to confirm this association.

## 6. Conclusion

SIRI is nonlinearly associated with the risk of AKI in ICU patients with AMI. Higher SIRI levels are an independent factor influencing the occurrence of AKI, and this association is more prominent in non-CKD patients compared to those with a history of CKD. Furthermore, SIRI is also an independent risk factor for in-hospital mortality in critically ill AMI patients and those with AMI complicated by AKI. This study confirms that SIRI is a clinically useful early predictor of AKI risk and in-hospital mortality in AMI patients, highlighting the crucial role of systemic inflammation.

## Figures and Tables

**Table 1:** Baseline characteristics of the AKI and non-AKI groups.

Variables	Total (*n* = 6936)	Without AKI (*n* = 1506)	AKI (*n* = 5430)	*p*
Age (years)	73.33 (18.14)	71.01 (20.08)	73.90 (17.38)	<0.001
Male (*n*, %)	4341 (62.59)	928 (61.62)	3413 (62.85)	0.381
White (*n*, %)	4652 (67.07)	1022 (67.86)	3630 (66.85)	0.460
BMI (kg/m^2^)	27.58 (7.43)	26.49 (6.45)	27.91 (7.63)	<0.001
T2DM (*n*, %)	2592 (37.37)	491 (32.60)	2101 (38.69)	<0.001
AF (*n*, %)	2572 (37.08)	378 (25.10)	2194 (40.41)	<0.001
AHF (*n*, %)	1973 (28.45)	263 (17.46)	1710 (31.49)	<0.001
STEMI (*n*, %)	3977 (57.34)	935 (62.08)	3042 (56.02)	<0.001
Hypertension (*n*, %)	2881 (41.54)	674 (44.75)	2207 (40.64)	0.004
CKD (*n*, %)	1562 (22.52)	259 (17.20)	1303 (24.00)	<0.001
CD (*n*, %)	489 (7.05)	100 (6.64)	389 (7.16)	0.482
CAG (*n*, %)	2444 (35.24)	669 (44.42)	1775 (32.69)	<0.001
CABG (*n*, %)	1854 (26.73)	303 (20.12)	1551 (28.56)	<0.001
PCI (*n*, %)	1576 (22.72)	533 (35.39)	1043 (19.21)	<0.001
Neutrophils (10^9^/L)	8.09 (5.84)	7.49 (5.15)	8.26 (6.01)	<0.001
TNT (µg/L)	0.45 (1.18)	0.46 (1.34)	0.45 (1.14)	0.256
CKMB (IU/L)	13.00 (35.00)	15.00 (46.08)	12.60 (32.47)	<0.001
Creatinine (mg/dL)	1.10 (0.80)	1.00 (0.50)	1.20 (0.80)	<0.001
Total bilirubin (mg/dL)	0.60 (0.50)	0.59 (0.40,)	0.60 (0.50)	<0.001
BUN (mg/dL)	22.00 (21.00)	19.00 (18.00)	23.00 (23.00)	<0.001
FBG (mg/dL)	137.00 (80.00)	129.00 (67.00)	139.00 (82.00)	<0.001
Albumin (g/dL)	3.40 (0.80)	3.60 (0.80)	3.30 (0.80)	<0.001
ALT (U/L)	29.00 (39.00)	29.00 (34.00)	29.00 (41.00)	0.106
AST (U/L)	43.00 (78.52)	41.00 (71.00)	44.00 (81.97)	0.010
Lactate (mmol/L)	1.79 (1.12)	1.82 (0.90)	1.70 (1.20)	0.312
UO (ml)	1760.00 (1532.00)	2320.00 (1533.75)	1610.00 (1410.00)	<0.001
SOFA	4.00 (5.00)	3.00 (4.00)	5.00 (5.00)	<0.001
ACEI/ARB (*n*, %)	3627 (52.29)	841 (55.84)	2786 (51.31)	0.002
*β*-Blockers (*n*, %)	5806 (83.71)	1271 (84.40)	4535 (83.52)	0.414
Insulin (*n*, %)	4979 (71.78)	856 (56.84)	4123 (75.93)	<0.001
Metformin (*n*, %)	402 (5.80)	85 (5.64)	317 (5.84)	0.776
SGLT2i (*n*, %)	35 (0.50)	9 (0.60)	26 (0.48)	0.565
Hospital Los Day (day)	8.81 (9.02)	5.65 (5.67)	9.86 (9.66)	<0.001
In-hospital mortality (*n*, %)	1060 (15.28)	79 (5.25)	981 (18.07)	<0.001

*Note:* Data are median (interquartile range), or *n* (%).

Abbreviations: ACEI/ARB, angiotensin-converting enzyme inhibitor/angiotensin receptor blocker; AF, atrial fibrillation; AHF, acute heart failure; ALT, alanine aminotransferase; AST, aspartate aminotransferase; BMI, body mass index; BUN, blood urea nitrogen; CABG, coronary artery bypass grafting; CAG, coronary angiography; CKD, chronic kidney disease; CKMB, creatine kinase MB; CD, cerebrovascular disease; FBG, fasting blood glucose; HP, hypertension; PCI, percutaneous coronary intervention; SGLT2i, sodium-glucose cotransporter 2 inhibitor; SOFA, sequential organ failure assessment; STEMI, ST-segment elevation myocardial infarction; T2DM, type 2 diabetes mellitus; TNT, troponin T; UO, urine output.

**Table 2:** Clinical outcomes according to SIRI quartiles.

Outcomes	Total (*n* = 6936)	Q1 (*n* = 1734)	Q2 (*n* = 1734)	Q3 (*n* = 1734)	Q4 (*n* = 1734)	*p*
Hospital Los Day (day)	8.81 (9.02)	8.08 (9.70)	8.09 (8.29)	9.07 (10.21)	9.88 (11.22)	<0.001
In-hospital mortality (*n*, %)	1060 (15.28)	142 (13.40)	157 (14.81)	286 (26.98)	475 (44.81)	<0.001
AKI (*n*, %)	5430 (78.29)	1252 (23.06)	1313 (24.18)	1388 (25.56)	1477 (27.20)	<0.001

*Note:* Data are median (interquartile range), or *n* (%). Hospital Los Day: hospital length of stay.

**Table 3:** Logistic regression analyses for primary endpoint.

SIRI	Model 1		Model 2		Model 3	
	OR (95% CI)	*p*	OR (95% CI)	*p*	OR (95% CI)	*p*
Q1	Ref	—	Ref	—	Ref	—
Q2	1.20 (1.03–1.40)	0.018	1.19 (1.02–1.39)	0.024	1.34 (1.13–1.60)	0.001
Q3	1.54 (1.32–1.81)	<0.001	1.53 (1.30–1.79)	<0.001	1.53 (1.28–1.84)	<0.001
Q4	2.21 (1.87–2.62)	<0.001	2.20 (1.86–2.61)	<0.001	1.76 (1.44–2.15)	<0.001

*Note*: Q1 ≤ 1.68; 1.68 < Q2 ≤ 3.51; 3.51 < Q3 ≤ 7.62; 7.62 < Q4, Model 1 was unadjusted, Model 2 was adjusted for sex, race, age, and BMI. Model 3 was adjusted for age, AF, AHF, CKD, ACEI/ARB, *β*-blockers, insulin, TNT, CKMB, albumin, UO, SOFA, CAG, CABG, and PCI.

Abbreviations: CI, confidence interval; OR, odds ratio.

**Table 4:** Logistic regression for primary outcome (excluding ICU stays <48 h).

SIRI	Model 1		Model 2		Model 3	
	OR (95% CI)	*p*	OR (95% CI)	*p*	OR (95% CI)	*p*
Q1	Ref	—	Ref	—	Ref	—
Q2	1.18 (1.01–1.38)	0.040	1.17 (0.99–1.37)	0.051	1.29 (1.08–1.54)	0.004
Q3	1.49 (1.26–1.75)	<0.001	1.47 (1.25–1.73)	<0.001	1.46 (1.21–1.76)	<0.001
Q4	2.19 (1.83–2.61)	<0.001	2.17 (1.82–2.59)	<0.001	1.70 (1.39–2.10)	<0.001

*Note:* Q1 ≤ 1.68; 1.68 < Q2 ≤ 3.48; 3.48 < Q3 ≤ 7.51; Q4 > 7.51. Model 1 was unadjusted. Model 2 was adjusted for sex, race, age, and BMI. Model 3 was adjusted for age, AF, AHF, CKD, ACEI/ARB, *β*-blockers, insulin, TNT, CKMB, albumin, UO, SOFA, CAG, CABG, and PCI.

Abbreviations: CI, confidence interval; OR, odds ratio.

**Table 5:** Conditional logistic regression analyses for primary outcome after PSM.

SIRI	Model 1		Model 2		Model 3	
	OR (95% CI)	*p*	OR (95% CI)	*p*	OR (95% CI)	*p*
<2.707	Ref	—	Ref	—	Ref	—
≥2.707	1.54 (1.33–1.79)	<0.001	1.53 (1.32–1.78)	<0.001	1.47 (1.26–1.72)	<0.001

*Note:* Model 1 was unadjusted. Model 2 was adjusted for sex, race, age, and BMI. Model 3 was adjusted for age, AF, AHF, CKD, ACEI/ARB, *β*-blockers, insulin, TNT, CKMB, albumin, UO, SOFA, CAG, CABG, and PCI.

Abbreviations: CI, confidence interval; OR, odds ratio.

**Table 6:** Cox regression for secondary outcome in the entire cohort.

SIRI	Model A		Model B		Model C	
	HR (95% CI)	*p*	HR (95% CI)	*p*	HR (95% CI)	*p*
Q1	Ref	—	Ref	—	Ref	—
Q2	1.04 (0.83–1.31)	0.722	1.04 (0.83–1.30)	0.739	1.03 (0.82–1.30)	0.773
Q3	1.66 (1.36–2.04)	<0.001	1.64 (1.34–2.00)	<0.001	1.40 (1.14–1.72)	0.001
Q4	2.58 (2.13–3.11)	<0.001	2.55 (2.12–3.08)	<0.001	1.74 (1.44–2.11)	<0.001

*Note:* Q1 ≤ 1.68; 1.68 < Q2 ≤ 3.51; 3.51 < Q3 ≤ 7.62; 7.62 < Q4. Model A was unadjusted. Model B was adjusted for sex, race, age, and BMI. Model C was adjusted for age, BMI, T2DM, AHF, CD, ACEI/ARB, SGLT2i, TNT, albumin, TB, FPG, ALT, AST, Lactate, UO and CAG.

Abbreviations: CI, confidence interval; HR, hazard ratio.

**Table 7:** Cox regression for secondary outcome, stratified by AKI status.

SIRI	With-AKI		Without-AKI	
	HR (95% CI)	*p*	HR (95% CI)	*p*
Q1	Ref	—	Ref	—
Q2	1.08 (0.85, 1.37)	0.527	0.51 (0.22, 1.20)	0.122
Q3	1.40 (1.13, 1.73)	0.002	1.25 (0.65, 2.43)	0.501
Q4	1.75 (1.43, 2.14)	<0.001	1.31 (0.68, 2.50)	0.414

*Note:* Q1 ≤ 1.68; 1.68 < Q2 ≤ 3.51; 3.51 < Q3 ≤ 7.62; 7.62 < Q4. Model C was adjusted for age, BMI, T2DM, AHF, CD, ACEI/ARB, SGLT2i, TNT, albumin, TB, FPG, ALT, AST, Lactate, UO and CAG.

Abbreviations: CI, confidence interval; HR, hazard ratio.

## Data Availability

The data that support the findings of this study are available from Massachusetts Institute of Technology but restrictions apply to the availability of these data, which were used under license for the current study, and so are not publicly available. The data are, however, available from the authors upon reasonable request and with permission of Massachusetts Institute of Technology.

## Nomenclature

AKI: Acute kidney injury
AMI: Acute myocardial infarction
ICU: Intensive care unit
SIRI: Systemic inflammation response index
AUC: Area under the receiver operating characteristic curve
TNF: Tumor necrosis factor-*α*
IL-1*β*: Interleukin-1*β*
IL-6: Interleukin-6
BIDMC: Beth Israel Deaconess Medical Center
MIT: Massachusetts Institute of Technology
AHF: Acute heart failure
AF: Atrial fibrillation
CKD: Chronic kidney disease
T2DM: Type 2 diabetes mellitus
CD: Cerebrovascular disease
ICD: International Classification of Diseases
SQL: Structured Query Language
PFC: Proportion of falsely classified
NRMSE: Normalized root mean square error
SOFA: Sequential organ failure assessment
Scr: Serum creatinine
KDIGO: Kidney Disease: Improving Global Outcomes
SD: Standard deviation
IQR: Interquartile range
ANOVA: One-way analysis of variance
MLR: Monocyte to lymphocyte ratio
NLR: Neutrophil to lymphocyte ratio
NMLR: Neutrophil to monocyte plus lymphocyte ratio
BMI: Body mass index
STEMIS: T-segment elevation myocardial infarction
CKD5: Stage 5 chronic kidney disease
ACEI: Angiotensin-converting enzyme inhibitors
ARB: Angiotensin receptor blockers
TB: Total bilirubin
BUN: Blood urea nitrogen
FBG: Fasting blood glucose
ALT: Alanine aminotransferase
AST: Aspartate aminotransferase
CAG: Coronary angiography
CABG: Coronary artery bypass grafting
PCI: Percutaneous coronary intervention
VIF: Variance inflation factor
OR: Odds ratio
CI: Confidence interval
RCS: Restricted cubic spline
DCA: Decision curve analysis
NRI: Net reclassification improvement
IDI: Integrated discrimination improvement
PSM: Propensity score matching
SMD: Standardized mean difference
TNT: Troponin T
CKMB: Creatine kinase MB
HR: Hazard ratio
UO: Urine output
NT-pro BNP: N-terminal pro B-type natriuretic peptide
SGLT2i: Sodium-glucose cotransporter 2 inhibitors
Hospital Los Day: Hospital length of stay
ACS: Acute coronary syndrome
CAD: Coronary artery disease
ROS: Reactive oxygen species
Th1: T helper 1
Th2: T helper 2
Tregs: Regulatory T cells
CRS: Cardiorenal syndrome
TG: Triglycerides
TC: Total cholesterol
HDL: High-density lipoprotein
LDL: Low-density lipoprotein
EF: Ejection fraction.

## Supporting Information

Supporting Information Table S1. Comparison of predictive performance for AKI among inflammatory indices. Table S2. Results of the multivariable logistic regression model for AKI prediction (Model 3). Table S3. Risk model comparison with and without SIRI. Table S4. Predictive performance of SIRI at different cutoff values for AKI. Table S5. Predictive characteristics of SIRI at Youden's index-derived cut point. Table S6. Baseline characteristics before and after propensity score matching. Table S7. Conditional logistic regression for primary outcome post-PSM based on SIRI quartiles from original cohort. Table S8. Results of the multivariable cox regression model for in-hospital mortality prediction (Model C). Table S9. Results of the multivariable cox regression model incorporating 48 h AKI status. Table S10. Logistic regression analyses of SIRI components for primary endpoint. Figure S1. Convergence of missForest imputation error across iterations. Normalized root mean square error (NRMSE) for continuous variables (A) and proportion of falsely classified (PFC) for categorical variables (B). Figure S2. Internal validation of Model 3 using bootstrap resampling. (A) Receiver operating characteristic (ROC) curve; (B) calibration curve; (C) decision curve analysis (DCA).
