# Supplementary material for: The Systemic Inflammation Response Index as an Independent Predictor of Acute Kidney Injury in Critically Ill Patients With Acute Myocardial Infarction: Insights From a Large-Scale Cohort Study
Source: Mediators Inflamm. 2025 Nov 21;2025:1417075. doi: 10.1155/mi/1417075 (PMC12662686; doi:10.1155/mi/1417075)
Supplement: Supporting Information — Table S1. Comparison of predictive performance for AKI among inflammatory indices. Table S2. Results of the multivariable logistic regression model for AKI prediction (Model 3). Table S3. Risk model comparison with and without SIRI. Table S4. Predictive performance of SIRI at different cutoff values for AKI. Table S5. Predictive characteristics of SIRI at Youden's index-derived cut point. Table S6. Baseline characteristics before and after propensity score matching. Table S7. Conditional logistic regression for primary outcome post-PSM based on SIRI quartiles from original cohort. Table S8. Results of the multivariable cox regression model for in-hospital mortality prediction (Model C). Table S9. Results of the multivariable cox regression model incorporating 48 h AKI status. Table S10. Logistic regression analyses of SIRI components for primary endpoint. Figure S1. Convergence of missForest imputation error across iterations. Normalized root mean square error (NRMSE) for continuous variables (A) and proportion of falsely classified (PFC) for categorical variables (B). Figure S2. Internal validation of Model 3 using bootstrap resampling. (A) Receiver operating characteristic (ROC) curve; (B) calibration curve; (C) decision curve analysis (DCA). [file 1417075.f1.docx]

Table S1 Comparison of predictive performance for AKI among inflammatory indices

| Variable | AUC | 95%CI | P |
| --- | --- | --- | --- |
| SIRI | 0.584 | (0.572,0.603) | Ref |
| MLR | 0.578 | (0.562,0.593) | 0.129 |
| NLR | 0.583 | (0.569,0.602) | 0.782 |
| NMLR | 0.583 | (0.570,0.603) | 0.669 |

AUC: area under the receiver operating characteristic curve, CI: confidence interval, MLR: monocyte to lymphocyte ratio, NLR: neutrophil to lymphocyte ratio, NMLR: neutrophil to monocyte plus lymphocyte ratio.

Table S2 Results of the multivariable logistic regression model for AKI prediction (Model 3)

| Variables | β | S.E | Z | *P* | OR (95%CI) | VIF |
| --- | --- | --- | --- | --- | --- | --- |
| Intercept | 1.13 | 0.25 | 4.42 | <.001 | 3.08 (1.87 ~ 5.08) |  |
| AF |  |  |  |  |  | 1.086 |
| 0 |  |  |  |  | 1.00 (Reference) |  |
| 1 | 0.38 | 0.07 | 5.21 | <.001 | 1.46 (1.26 ~ 1.68) |  |
| AHF |  |  |  |  |  | 1.088 |
| 0 |  |  |  |  | 1.00 (Reference) |  |
| 1 | 0.76 | 0.08 | 9.18 | <.001 | 2.13 (1.81 ~ 2.51) |  |
| CKD |  |  |  |  |  | 1.110 |
| 0 |  |  |  |  | 1.00 (Reference) |  |
| 1 | 0.01 | 0.09 | 0.13 | 0.899 | 1.01 (0.85 ~ 1.20) |  |
| ACEI/ARB |  |  |  |  |  | 1.205 |
| 0 |  |  |  |  | 1.00 (Reference) |  |
| 1 | 0.26 | 0.07 | 3.68 | <.001 | 1.30 (1.13 ~ 1.49) |  |
| Β-blockers |  |  |  |  |  | 1.220 |
| 0 |  |  |  |  | 1.00 (Reference) |  |
| 1 | 0.24 | 0.10 | 2.47 | 0.013 | 1.27 (1.05 ~ 1.54) |  |
| PCI |  |  |  |  |  | 2.227 |
| 0 |  |  |  |  | 1.00 (Reference) |  |
| 1 | -0.36 | 0.11 | -3.25 | 0.001 | 0.70 (0.56 ~ 0.87) |  |
| CAG |  |  |  |  |  | 1.936 |
| 0 |  |  |  |  | 1.00 (Reference) |  |
| 1 | 0.29 | 0.10 | 2.94 | 0.003 | 1.33 (1.10 ~ 1.61) |  |
| CABG |  |  |  |  |  | 1.677 |
| 0 |  |  |  |  | 1.00 (Reference) |  |
| 1 | 0.65 | 0.10 | 6.47 | <.001 | 1.91 (1.57 ~ 2.32) |  |
| Insulin |  |  |  |  |  | 1.237 |
| 0 |  |  |  |  | 1.00 (Reference) |  |
| 1 | 0.58 | 0.08 | 7.67 | <.001 | 1.78 (1.54 ~ 2.06) |  |
| TNT | 0.01 | 0.02 | 0.70 | 0.482 | 1.01 (0.98 ~ 1.05) | 2.208 |
| CKMB | 0.00 | 0.00 | 1.85 | 0.064 | 1.00 (1.00 ~ 1.00) | 2.218 |
| Albumin | -0.43 | 0.06 | -7.02 | <.001 | 0.65 (0.57 ~ 0.73) | 1.376 |
| UO | -0.01 | 0.00 | -12.47 | <.001 | 0.99 (0.99 ~ 0.99) | 1.105 |
| SOFA | 0.17 | 0.01 | 13.46 | <.001 | 1.19 (1.16 ~ 1.22) | 1.328 |
| SIRI |  |  |  |  |  | 1.246 |
| Q1 |  |  |  |  | 1.00 (Reference) |  |
| Q2 | 0.30 | 0.09 | 3.39 | 0.001 | 1.34 (1.13 ~ 1.60) |  |
| Q3 | 0.43 | 0.09 | 4.62 | <.001 | 1.53 (1.28 ~ 1.84) |  |
| Q4 | 0.57 | 0.10 | 5.56 | <.001 | 1.76 (1.44 ~ 2.15) |  |

OR: Odds Ratio, CI: Confidence Interval
SIRI: Q1:<=1.68;1.68<Q2<=3.51;3.51<Q3<=7.62;7.62<Q4
Model 3 was adjusted for age, AF, AHF, CKD, ACEI/ARB, β-blockers, insulin, TNT, CKMB, albumin, UO, SOFA, CAG, CABG, and PCI.

Table S3 Risk model comparison with and without SIRI

|  | Value | 95%CI | P |
| --- | --- | --- | --- |
| Continuous NRI | 19.84% | (14.16%, 25.53%) | <0.001 |
| IDI | 0.54% | (0.34%, 0.73%) | <0.001 |

NRI: Net Reclassification Improvement, IDI: Integrated Discrimination Improvement

Table S4 Predictive performance of SIRI at different cutoff values for AKI

| Cutoff | Sensitivity | Specificity | Notes |
| --- | --- | --- | --- |
| 0.000 | .997 | .003 |  |
| 0.586 | .950 | .068 | High Sensitivity |
| 0.887 | .900 | .126 |  |
| 1.181 | .850 | .203 |  |
| 1.475 | .800 | .284 |  |
| 2.707 | .624 | .513 |  |
| 6.674 | .311 | .800 |  |
| 8.365 | .247 | .850 |  |
| 11.644 | .170 | .900 |  |
| 19.307 | .086 | .950 | High Specificity |
| 565.922 | .000 | 1.000 |  |

Table S5 Predictive characteristics of SIRI at Youden's Index-derived cut-point

| AUC (95%CI) | Accuracy (95%CI) | Sensitivity (95%CI) | Specificity (95%CI) | PPV (95%CI) | NPV (95%CI) | Cut off |
| --- | --- | --- | --- | --- | --- | --- |
|  |  |  |  |  |  |  |
| 0.58 (0.57-0.60) | 0.60 (0.59-0.61) | 0.51 (0.49 - 0.54) | 0.62 (0.61 - 0.64) | 0.27 (0.26 - 0.29) | 0.82 (0.81 - 0.83) | 2.707 |

Table S6 Baseline characteristics before and after propensity score matching

| Variable | Before PSM | | | | | | | After PSM | | | | |
| --- | --- | --- | --- | --- | --- | --- | --- | --- | --- | --- | --- | --- |
|  | Total (n = 6936) | 0 (n = 2814) | 1 (n = 4122) | *P* | SMD |  | Total (n = 4242) | | 0 (n = 2121) | 1 (n = 2121) | *P* | SMD |
| Age, (years) | 73.33 (18.14) | 71.30 (18.09) | 74.56 (17.65) | <.001 | 0.119 |  | 73.24 (17.95) | | 72.76 (18.44) | 73.64 (17.44) | 0.124 | 0.002 |
| Male, (n, %) | 4341 (62.59) | 1775 (63.08) | 2566 (62.25) | 0.485 | 0.017 |  | 2610 (61.53) | | 1279 (60.30) | 1331 (62.75) | 0.101 | 0.051 |
| White, (n, %) | 4652 (67.07) | 1854 (65.88) | 2798 (67.88) | 0.083 | 0.043 |  | 2825 (66.6) | | 1424 (67.14) | 1401 (66.05) | 0.454 | 0.023 |
| BMI, kg/m^2^ | 27.58 (7.43) | 27.68 (6.91) | 27.51 (7.71) | 0.065 | 0.009 |  | 27.61 (7.32) | | 27.49 (7.08) | 27.69 (7.48) | 0.167 | 0.004 |
| TNT, (µg/L) | 0.45 (1.18) | 0.40 (0.91) | 0.51 (1.41) | <.001 | 0.186 |  | 0.46 (1.17) | | 0.45 (1.14) | 0.49 (1.22) | 0.162 | 0.033 |
| CKMB, (IU/L) | 13.00 (35.00) | 11.00 (26.00) | 15.00 (42.75) | <.001 | 0.182 |  | 13.45 (36.00) | | 12.80 (33.00) | 15.00 (38.00) | 0.024 | 0.057 |
| Creatinine, (mg/dL) | 1.10 (0.80) | 1.00 (0.60) | 1.20 (0.90) | <.001 | 0.213 |  | 1.10 (0.70) | | 1.10 (0.70) | 1.10 (0.80) | 0.047 | 0.032 |
| Total Bilirubin, (mg/dL) | 0.60 (0.50) | 0.50 (0.40) | 0.60 (0.50) | <.001 | 0.138 |  | 0.60 (0.40) | | 0.60 (0.40) | 0.60 (0.42) | 0.003 | 0.042 |
| BUN, (mg/dL) | 22.00 (21.00) | 19.00 (15.00) | 25.00 (25.00) | <.001 | 0.337 |  | 21.00 (20.00) | | 21.00 (19.00) | 22.00 (20.00) | <.001 | 0.077 |
| FBG, (mg/dL) (Q₁, Q₃) | 137.00 (80.00) | 126.00 (68.00) | 144.00 (85.00) | <.001 | 0.216 |  | 134.00 (77.00) | | 129.00 (75.00) | 139.00 (76.00) | <.001 | 0.057 |
| Albumin, (g/dL) | 3.40 (0.80) | 3.60 (0.80) | 3.20 (0.80) | <.001 | -0.537 |  | 3.44 (0.79) | | 3.50 (0.74) | 3.40 (0.80) | 0.009 | -0.053 |
| ALT, (U/L) | 29.00 (39.00) | 26.00 (26.38) | 33.00 (51.89) | <.001 | 0.113 |  | 28.00 (34.00) | | 27.00 (31.00) | 30.00 (38.00) | <.001 | 0.025 |
| AST, (U/L) | 43.00 (78.52) | 34.00 (47.00) | 54.00 (109.01) | <.001 | 0.116 |  | 42.00 (72.00) | | 38.23 (60.00) | 47.00 (85.00) | <.001 | 0.023 |
| Lactate, (mmol/L) | 1.79 (1.12) | 1.60 (0.90) | 1.90 (1.30) | <.001 | 0.204 |  | 1.75 (1.08) | | 1.70 (0.93) | 1.80 (1.20) | <.001 | 0.047 |
| UO, (ml) | 1760.00 (1532.00) | 1950.00 (1475.00) | 1615.00 (1520.00) | <.001 | -0.173 |  | 1795.00 (1576.00) | | 1840.00 (1550.00) | 1750.00 (1560.00) | 0.109 | -0.007 |
| SOFA | 4.00 (5.00) | 4.00 (4.00) | 5.00 (5.00) | <.001 | 0.215 |  | 4.00 (5.00) | | 4.00 (4.00) | 4.00 (5.00) | 0.004 | 0.059 |
| T2DM, (n, %) | 2592 (37.37) | 1112 (39.52) | 1480 (35.90) | 0.002 | 0.075 |  | 1588 (37.44) | | 792 (37.34) | 796 (37.53) | 0.899 | 0.004 |
| AF, (n, %) | 2572 (37.08) | 891 (31.66) | 1681 (40.78) | <.001 | 0.186 |  | 1474 (34.75) | | 725 (34.18) | 749 (35.31) | 0.439 | 0.024 |
| AHF, (n, %) | 1973 (28.45) | 624 (22.17) | 1349 (32.73) | <.001 | 0.225 |  | 1159 (27.32) | | 568 (26.78) | 591 (27.86) | 0.428 | 0.024 |
| STEMI, (n, %) | 3977 (57.34) | 1622 (57.64) | 2355 (57.13) | 0.675 | 0.010 |  | 2563 (60.42) | | 1332 (62.80) | 1231 (58.04) | 0.002 | 0.096 |
| Hypertension, (n, %) | 2881 (41.54) | 1357 (48.22) | 1524 (36.97) | <.001 | 0.233 |  | 1842 (43.42) | | 923 (43.52) | 919 (43.33) | 0.901 | 0.004 |
| CKD, (n, %) | 1562 (22.52) | 567 (20.15) | 995 (24.14) | <.001 | 0.093 |  | 930 (21.92) | | 461 (21.74) | 469 (22.11) | 0.767 | 0.009 |
| CVD, (n, %) | 489 (7.05) | 207 (7.36) | 282 (6.84) | 0.411 | 0.020 |  | 311 (7.33) | | 163 (7.69) | 148 (6.98) | 0.377 | 0.028 |
| ACEI/ARB, (n, %) | 3627 (52.29) | 1561 (55.47) | 2066 (50.12) | <.001 | 0.107 |  | 2332 (54.97) | | 1177 (55.49) | 1155 (54.46) | 0.497 | 0.021 |
| β blockers, (n, %) | 5806 (83.71) | 2491 (88.52) | 3315 (80.42) | <.001 | 0.204 |  | 3570 (84.16) | | 1803 (85.01) | 1767 (83.31) | 0.130 | 0.046 |
| Insulin, (n, %) | 4979 (71.78) | 2131 (75.73) | 2848 (69.09) | <.001 | 0.144 |  | 2965 (69.9) | | 1463 (68.98) | 1502 (70.82) | 0.192 | 0.040 |
| Metformin, (n, %) | 402 (5.8) | 277 (9.84) | 125 (3.03) | <.001 | 0.397 |  | 205 (4.83) | | 97 (4.57) | 108 (5.09) | 0.431 | 0.024 |
| SGLT2i, (n, %) | 35 (0.5) | 12 (0.43) | 23 (0.56) | 0.448 | 0.018 |  | 17 (0.4) | | 9 (0.42) | 8 (0.38) | 0.808 | 0.008 |
| CAG, (n, %) | 2444 (35.24) | 962 (34.19) | 1482 (35.95) | 0.130 | 0.037 |  | 1664 (39.23) | | 852 (40.17) | 812 (38.28) | 0.208 | 0.039 |
| CABG, (n, %) | 1854 (26.73) | 1230 (43.71) | 624 (15.14) | <.001 | 0.797 |  | 1131 (26.66) | | 570 (26.87) | 561 (26.45) | 0.755 | 0.010 |
| PCI, (n, %) | 1576 (22.72) | 616 (21.89) | 960 (23.29) | 0.172 | 0.033 |  | 1095 (25.81) | | 570 (26.87) | 525 (24.75) | 0.114 | 0.049 |

Data are median (interquartile range), or n (%) BMI: body mass index, T2DM: type 2 diabetes mellitus, AF: atrial fibrillation, AHF: acute heart failure, STEMI : ST-segment elevation myocardial infarction, HP: Hypertension, CKD: chronic kidney disease, CVD: cerebrovascular disease, CAG: coronary angiography, CABG: coronary artery bypass grafting, PCI : percutaneous coronary intervention, SOFA :sequential organ failure assessment, TNT: Troponin T, CKMB : creatine kinase MB, BUN: blood urea nitrogen, FBG: fasting blood glucose, ALT: alanine aminotransferase, AST: aspartate aminotransferase, UO: Urine output, SOFA: sequential organ failure assessment, ACEI/ARB : angiotensin-converting enzyme inhibitor/ angiotensin receptor blocker, SGLT2i: sodium-glucose cotransporter 2 inhibitor.

Table S7 Conditional logistic regression for primary outcome post-PSM based on SIRI quartiles from original cohort

| SIRI (n) | Model 1 | | Model 2 | | Model 3 | |  |
| --- | --- | --- | --- | --- | --- | --- | --- |
|  | OR (95%CI) | P | OR (95%CI) | P | OR (95%CI) | P |  |
| Q1(1237) | | Ref |  | Ref |  | Ref |  |
| Q2(1296) | | 1.25 (0.97 - 1.61) | 0.091 | 1.25 (0.97 - 1.62) | 0.085 | 1.37 (1.12 - 1.67) | 0.02 |
| Q3(957) | | 1.41 (1.11 - 1.79) | 0.005 | 1.40 (1.10 - 1.78) | 0.006 | 1.48 (1.19 - 1.84) | <.001 |
| Q4(752) | | 2.17 (1.63 - 2.90) | <.001 | 2.23 (1.67 - 2.98) | <.001 | 1.78 (1.38 - 2.29) | <.001 |

OR: Odds Ratio, CI: Confidence Interval
Q1:<=1.68;1.68<Q2<=3.51;3.51<Q3<=7.62;7.62<Q4
Model 1 was unadjusted
Model 2 was adjusted for sex, race, age, and BMI.
Model 3 was adjusted for age, AF, AHF, CKD, ACEI/ARB, β-blockers, insulin, TNT, CKMB, albumin, UO, SOFA, CAG, CABG, and PCI

Table S8 Results of the multivariable cox regression model for In-hospital mortality prediction (Model C)

| Variables | β | S.E | Z | *P* | HR (95%CI) | VIF |
| --- | --- | --- | --- | --- | --- | --- |
| AHF |  |  |  |  |  | 1.032 |
| 0 |  |  |  |  | 1.00 (Reference) |  |
| 1 | -0.17 | 0.07 | -2.45 | 0.014 | 0.84 (0.73 ~ 0.97) |  |
| T2DM |  |  |  |  |  | 1.122 |
| 0 |  |  |  |  | 1.00 (Reference) |  |
| 1 | -0.06 | 0.07 | -0.94 | 0.346 | 0.94 (0.82 ~ 1.07) |  |
| CD |  |  |  |  |  | 1.010 |
| 0 |  |  |  |  | 1.00 (Reference) |  |
| 1 | 0.24 | 0.11 | 2.09 | 0.037 | 1.27 (1.01 ~ 1.58) |  |
| ACEIARB |  |  |  |  |  | 1.134 |
| 0 |  |  |  |  | 1.00 (Reference) |  |
| 1 | -1.05 | 0.08 | -14.04 | <.001 | 0.35 (0.30 ~ 0.40) |  |
| SGLT2i |  |  |  |  |  | 1.010 |
| 0 |  |  |  |  | 1.00 (Reference) |  |
| 1 | -0.83 | 0.71 | -1.16 | 0.245 | 0.44 (0.11 ~ 1.76) |  |
| CAG |  |  |  |  |  | 1.221 |
| 0 |  |  |  |  | 1.00 (Reference) |  |
| 1 | 0.11 | 0.08 | 1.37 | 0.171 | 1.11 (0.95 ~ 1.30) |  |
| Age | 0.01 | 0.00 | 6.38 | <.001 | 1.01 (1.01 ~ 1.01) | 1.028 |
| BMI | -0.00 | 0.00 | -0.48 | 0.633 | 1.00 (0.99 ~ 1.00) | 1.002 |
| TNT | 0.06 | 0.01 | 6.81 | <.001 | 1.07 (1.05 ~ 1.08) | 1.164 |
| Albumin | -0.39 | 0.05 | -7.43 | <.001 | 0.68 (0.61 ~ 0.75) | 1.202 |
| Total Bilirubin | 0.01 | 0.01 | 1.50 | 0.133 | 1.01 (1.00 ~ 1.03) | 1.046 |
| FBG | 0.01 | 0.00 | 3.60 | <.001 | 1.01 (1.01 ~ 1.01) | 1.200 |
| ALT | 0.00 | 0.00 | 0.75 | 0.456 | 1.00 (1.00 ~ 1.00) | 4.480 |
| AST | -0.01 | 0.00 | -1.98 | 0.048 | 0.99 (0.99 ~ 0.99) | 4.520 |
| Lactate | 0.12 | 0.01 | 12.60 | <.001 | 1.13 (1.11 ~ 1.15) | 1.188 |
| UO | -0.01 | 0.00 | -7.99 | <.001 | 0.99 (0.99 ~ 0.99) | 1.089 |
| SIRI |  |  |  |  |  | 1.157 |
| Q1 |  |  |  |  | 1.00 (Reference) |  |
| Q2 | 0.03 | 0.12 | 0.29 | 0.773 | 1.03 (0.82 ~ 1.30) |  |
| Q3 | 0.34 | 0.10 | 3.22 | 0.001 | 1.40 (1.14 ~ 1.72) |  |
| Q4 | 0.55 | 0.10 | 5.67 | <.001 | 1.74 (1.44 ~ 2.11) |  |

HR: Hazard Ratio, CI: Confidence Interval
Q1:<=1.68;1.68<Q2<=3.51;3.51<Q3<=7.62;7.62<Q4
Model C was adjusted for age, BMI, T2DM, AHF, CD, ACEI/ARB, SGLT2i, TNT, albumin, TB, FPG, ALT, AST, Lactate, UO and CAG.

Table S9 Results of the multivariable cox regression model incorporating 48-hour AKI status

| Variables | β | S.E | Z | *P* | HR (95%CI) |
| --- | --- | --- | --- | --- | --- |
|  |  |  |  |  |  |
| T2DM |  |  |  |  |  |
| 0 |  |  |  |  | 1.00 (Reference) |
| 1 | -0.08 | 0.08 | -1.09 | 0.274 | 0.92 (0.79 ~ 1.07) |
| AHF |  |  |  |  |  |
| 0 |  |  |  |  | 1.00 (Reference) |
| 1 | -0.11 | 0.08 | -1.53 | 0.127 | 0.89 (0.77 ~ 1.03) |
| CD |  |  |  |  |  |
| 0 |  |  |  |  | 1.00 (Reference) |
| 1 | 0.30 | 0.12 | 2.53 | 0.012 | 1.35 (1.07 ~ 1.71) |
| ACEIARB |  |  |  |  |  |
| 0 |  |  |  |  | 1.00 (Reference) |
| 1 | -0.90 | 0.08 | -11.44 | <.001 | 0.41 (0.35 ~ 0.48) |
| SGLT2i |  |  |  |  |  |
| 0 |  |  |  |  | 1.00 (Reference) |
| 1 | -0.68 | 0.71 | -0.95 | 0.342 | 0.51 (0.13 ~ 2.05) |
| CAG |  |  |  |  |  |
| 0 |  |  |  |  | 1.00 (Reference) |
| 1 | 0.08 | 0.09 | 0.98 | 0.330 | 1.09 (0.92 ~ 1.29) |
| Age | 0.01 | 0.00 | 6.31 | <.001 | 1.01 (1.01 ~ 1.01) |
| BMI | -0.00 | 0.00 | -0.76 | 0.449 | 1.00 (0.99 ~ 1.01) |
| Total Bilirubin | 0.01 | 0.01 | 1.23 | 0.218 | 1.01 (0.99 ~ 1.03) |
| TNT | 0.04 | 0.01 | 3.65 | <.001 | 1.05 (1.02 ~ 1.07) |
| Albumin | -0.37 | 0.06 | -6.53 | <.001 | 0.69 (0.62 ~ 0.77) |
| FBG | 0.01 | 0.00 | 2.37 | 0.018 | 1.01 (1.01 ~ 1.01) |
| ALT | 0.00 | 0.00 | 1.57 | 0.117 | 1.00 (1.00 ~ 1.00) |
| AST | -0.01 | 0.00 | -2.48 | 0.013 | 0.99 (0.99 ~ 0.99) |
| UO | -0.01 | 0.00 | -5.08 | <.001 | 0.99 (0.99 ~ 0.99) |
| Lactate | 0.09 | 0.01 | 6.64 | <.001 | 1.09 (1.06 ~ 1.12) |
| SIRI |  |  |  |  |  |
| Q1 |  |  |  |  | 1.00 (Reference) |
| Q2 | 0.02 | 0.13 | 0.13 | 0.898 | 1.02 (0.79 ~ 1.31) |
| Q3 | 0.32 | 0.11 | 2.83 | 0.005 | 1.38 (1.10 ~ 1.73) |
| Q4 | 0.56 | 0.11 | 5.18 | <.001 | 1.75 (1.41 ~ 2.16) |
| AKI |  |  |  |  |  |
| 0 |  |  |  |  | 1.00 (Reference) |
| 1 | 0.80 | 0.17 | 4.60 | <.001 | 2.22 (1.58 ~ 3.12) |
| HR: Hazard Ratio, CI: Confidence Interval | | | | | |

Table S10 Logistic regression analyses of SIRI components for primary endpoint

| Variables | β | S.E | Z | *P* | OR (95%CI) |
| --- | --- | --- | --- | --- | --- |
|  |  |  |  |  |  |
| Neutrophils | 0.03 | 0.01 | 5.98 | <.001 | 1.04 (1.02 ~ 1.05) |
| Lymphocyte | -0.04 | 0.01 | -2.58 | 0.010 | 0.96 (0.94 ~ 0.99) |
| Monocyte | 0.26 | 0.06 | 4.23 | <.001 | 1.30 (1.15 ~ 1.46) |
| OR: Odds Ratio, CI: Confidence Interval | | | | | |


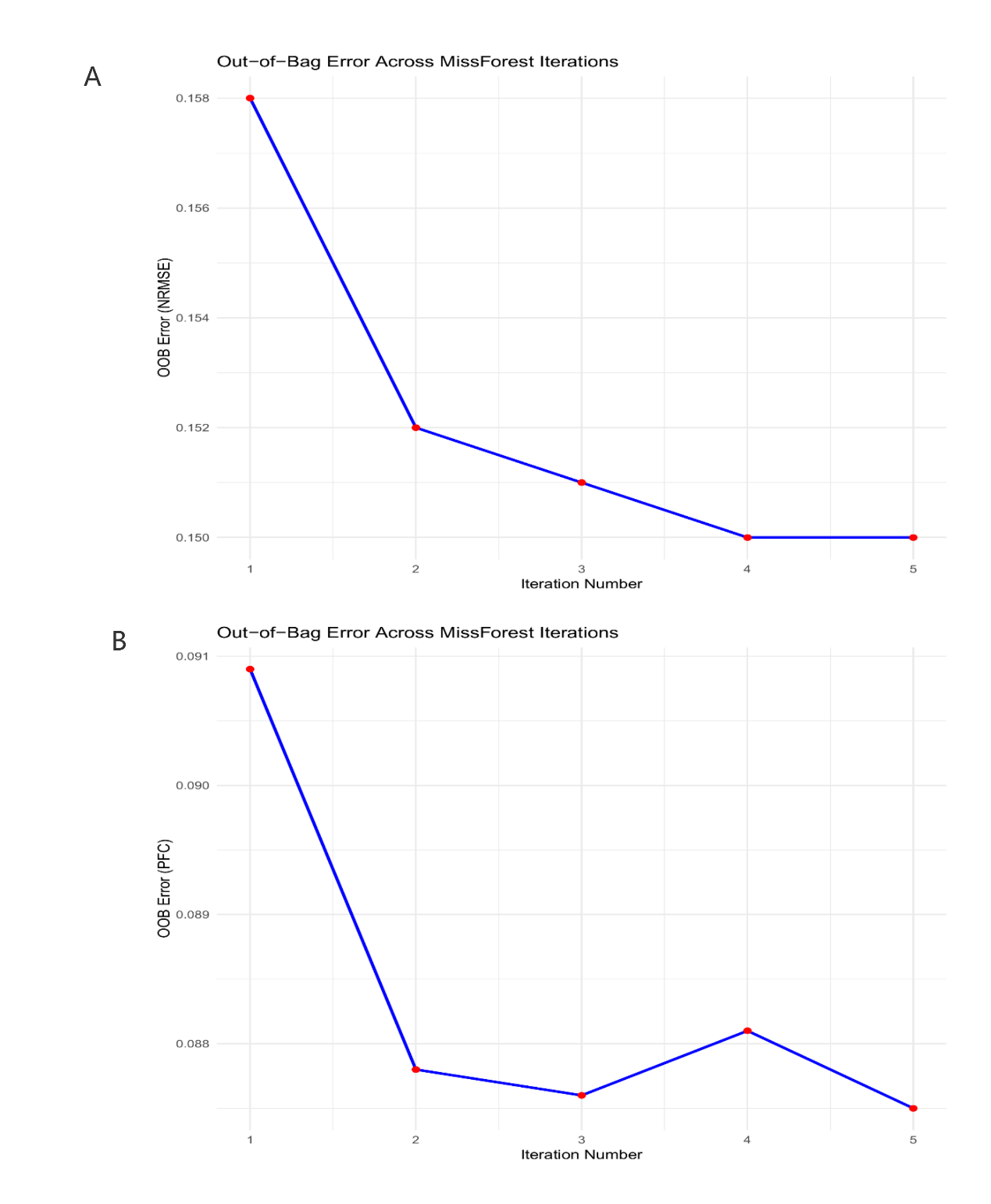


Fig.S1 Convergence of missForest imputation error across iterations. Normalized root mean square error (NRMSE) for continuous variables (A) and proportion of falsely classified (PFC) for categorical variables (B).


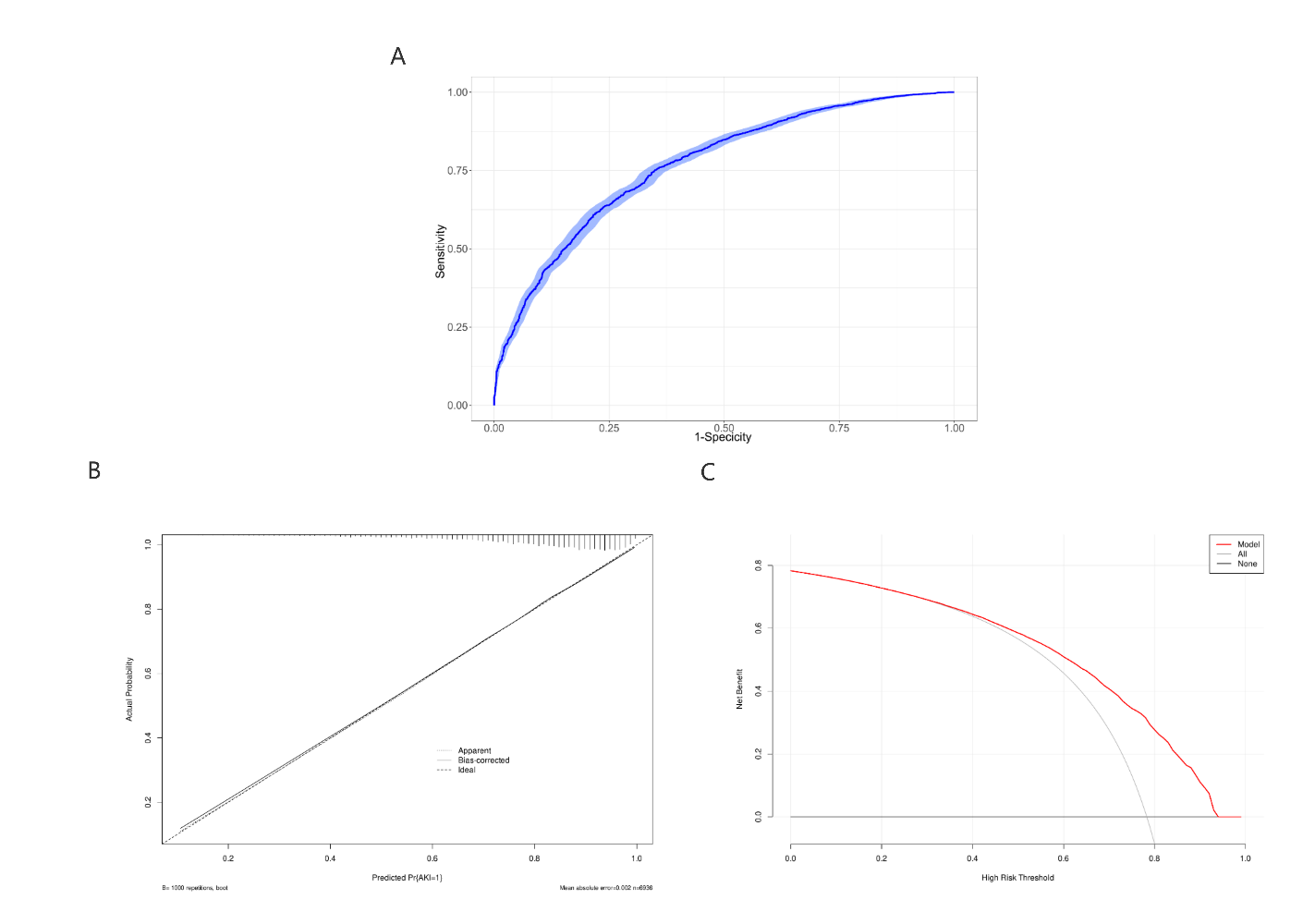


Fig S2. Internal Validation of Model 3 Using Bootstrap Resampling. (A) Receiver operating characteristic (ROC) curve; (B) Calibration curve; (C) Decision curve analysis (DCA).
